# Recruiting Pregnant Patients for Survey Research: A Head to Head Comparison of Social Media-Based Versus Clinic-Based Approaches

**DOI:** 10.2196/jmir.6593

**Published:** 2016-12-21

**Authors:** Lindsay Admon, Jessica K Haefner, Giselle E Kolenic, Tammy Chang, Matthew M Davis, Michelle H Moniz

**Affiliations:** ^1^ National Clinician Scholars Program University of Michigan Institute for Healthcare Policy and Innovation and the US Department of Veterans Affairs Ann Arbor, MI United States; ^2^ Department of Obstetrics and Gynecology University of Michigan Ann Arbor, MI United States; ^3^ Institute for Healthcare Policy and Innovation University of Michigan Ann Arbor, MI United States; ^4^ Department of Family Medicine University of Michigan Ann Arbor, MI United States; ^5^ Department of Pediatrics Northwestern Feinberg School of Medicine Chicago, IL United States

**Keywords:** pregnant women, surveys and questionnaires, methods, social media

## Abstract

**Background:**

Recruiting a diverse sample of pregnant women for clinical research is a challenging but crucial task for improving obstetric services and maternal and child health outcomes.

**Objective:**

To compare the feasibility and cost of recruiting pregnant women for survey research using social media-based and clinic-based approaches.

**Methods:**

Advertisements were used to recruit pregnant women from the social media website Facebook. In-person methods were used to recruit pregnant women from the outpatient clinic of a large, tertiary care center. In both approaches, potential respondents were invited to participate in a 15-minute Web-based survey. Each recruitment method was monitored for 1 month. Using bivariate statistics, we compared the number, demographic characteristics, and health characteristics of women recruited and the cost per completed survey for each recruitment method.

**Results:**

The social media-based approach recruited 1178 women and the clinic-based approach recruited 219 women. A higher proportion of subjects recruited through social media identified as African American (29.4%, 207/705 vs 11.2%, 20/179), reported household incomes <US $30,000 per year (56.8%, 409/720 vs 25.8%, 47/182), reported being in early pregnancy (18.6%, 135/726 vs 10.4%, 19/183 first trimester), and rated their health as fair or poor (22.2%, 160/722 vs 8.2%, 15/183; all *P*<.001). A smaller proportion of subjects recruited through social media had earned a college degree (21.3%, 153/717 vs 62.3%, 114/183) and were married or in a domestic partnership (45.7%, 330/722 vs 72.1%, 132/183; all *P*<.001). Social media-based recruitment costs were US $14.63 per completed survey, compared with US $23.51 for clinic-based recruitment.

**Conclusions:**

Web-based recruitment through a social networking platform is a feasible, inexpensive, and rapid means of recruiting a large, diverse sample of pregnant women for survey research.

## Introduction

Recruiting pregnant women for clinical research is a challenging but crucial task for improving obstetric services and maternal and child health outcomes. Conventional methods for recruiting pregnant women for survey research face significant limitations. Traditionally, pregnant women have been recruited from clinical care sites, an approach that is often plagued by underrepresentation of women in early pregnancy, poor demographic diversity, and the inability to access women who do not seek prenatal care—resulting in limited generalizability of study findings and inferential errors and bias in the use of such data [1,2]. Recruitment through established national samples (eg, Survey Sampling International, Gesellschaft fur Konsumforschung or GfK) is attractive for its methodological rigor and geographic diversity of participants. However, these companies are often only able to provide small samples of pregnant women, which limit analytic power. As a result, innovative strategies are needed to conduct methodologically sound and cost-effective survey research influencing the care of pregnant women.

The Internet and social media platforms offer promising avenues for recruitment of pregnant women. Recent data from the Pew Internet & American Life Project reveal that 96% of 18- to 29-year-olds in the United States have access to the Internet and that Internet use continues to rise among the adult population [3]. An even more recent phenomenon is the rise in popularity of social networking websites, with Facebook (Facebook Inc., Palo Alto, CA, USA) second only to Google (Google Inc., Mountain View, CA, USA) as the most popular website in the United States [4]. With 1.09 billion active users daily, Facebook has been used as an effective method to recruit female adolescents and women for survey research [1,5-15]. Little is known, however, about whether social media platforms are a feasible method for recruitment of pregnant women and how they compare with traditional recruitment methods in terms of cost.

Our team conducted a head-to-head comparison of 2 recruitment methods among pregnant women: Web-based recruitment through social media and traditional in-person recruitment at prenatal clinics. Our objective was to describe the relative advantages and disadvantages of each approach, including the total number of women recruited, their demographic and health characteristics, and the cost per completed survey for each recruitment method.

## Methods

### Recruitment

We utilized a cross-sectional design to invite respondents recruited via both methods to watch a brief Web-based video about health in pregnancy and answer survey items about pregnancy-related health knowledge and behaviors. Approximately 1 month of time was allocated to each recruitment method. Women were eligible for participation if they self-identified as being 18 years of age or older, pregnant, English speaking, and living in the United States. The study was deemed exempt by the study site’s institutional review board.

#### Recruitment of the Social Media-Based Sample

We recruited participants through advertisements on Facebook. Ads created on Facebook contained three key features: an image, a caption, and an ad copy containing a link to the survey website. Study ads were developed by the investigators using Facebook’s self-service application. Ads were shown on Facebook users’ newsfeeds. Clicking on the hyperlink within the ad led users to our survey website.

Facebook’s platform can direct ads to specific audiences based on sex, age, location, and interests. Interest-based targeting parameters such as “expectant parents,” “interest in motherhood,” and “parents”—or a combination of these—may be utilized. Facebook uses a proprietary method to infer users’ racial and ethnic “interests” by analyzing pages and posts users have liked or engaged with on Facebook. These inferences can be used as targeting parameters. Ad content can also be tailored. For example, ad images may differ in the race of women depicted.

Before study recruitment began, ads were refined over 11 days during May 2015 by testing a variety of combinations of image, caption, ad copy, and interest-based targeting parameters. During this ad refinement phase, Facebook automatically monitored the cost per click on the survey link included in each potential recruitment ad, as well as the cost per completed survey. This enabled the study team to learn which ads worked best for progressive outreach to audiences of particular interest. To investigate the effects of financial incentives on recruitment, US $5 and US $10 incentives were tested during this period, with results showing that the US $10 offer attracted more clicks. Availability of incentives was indicated in the ads that appeared on Facebook. Respondents who completed the survey during the ad refinement period, 21 in total, are not included in the recruitment numbers.

**Figure 1:**
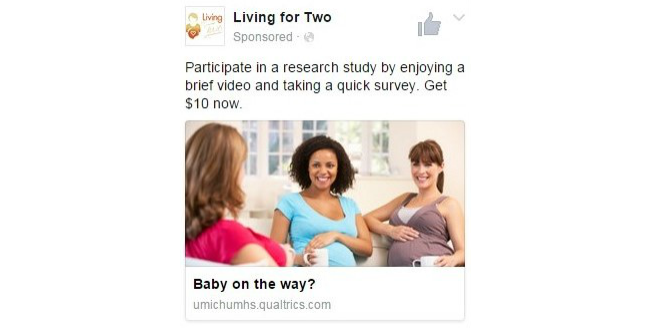
Sample Facebook advertisement used for social media–based recruitment of pregnant women.

The final campaign included the highest-performing ads with US $10 incentives, ultimately targeting women aged 18 years and older, living in the United States, and fitting the following interest profiles: African American expectant parents, Asian American expectant parents, and Hispanic women interested in motherhood. A sample Facebook ad is provided in Figure 1.

Following pilot testing, women were recruited through Facebook during 27 consecutive days in May-June 2015 utilizing the targeting parameters listed above. Potential participants were able to engage with the study materials via their own Internet access (mobile devices, personal computers, public computers, etc).

#### Recruitment of the Clinical Sample

Clinic-based recruitment occurred over 29 business days in August-September 2015. Pregnant women were recruited at routine outpatient obstetric visits at a large tertiary care center in the Midwest. Clinic clerks distributed study fliers to all patients presenting for prenatal appointments. Patients who expressed interest in participating were directed to trained research staff in the waiting room. Research staff invited participation, assessed eligibility, and answered questions about the study based on a standardized recruitment script. Patients completed the survey in the clinic waiting room or examination room using a tablet and headphones provided by the study team. Research staff sent participants individual survey links by email if they preferred to take the survey via their own Internet access. No identifying information was collected, and survey responses were not linked to participants’ clinical information. Because of the high proportion of women interested in participation with the US $5 incentive, US $10 incentives were not offered.

### Survey Completion

The survey’s introductory webpage explained the purpose of the study, the anonymous nature of the research, the expected time needed to complete the survey (15 minutes), and the option to quit at any time. Upon completion of the survey, participants were asked to provide their email address for delivery of the incentive. Participant email addresses were reviewed for repetition or similarity to exclude duplicate respondents who appeared to be seeking multiple incentives.

### Statistical Analysis

All data were deidentified and analyzed in Stata 14 (StataCorp LP).

#### Demographics

Demographic information and health characteristics were first investigated with descriptive statistics, including means and proportions, and stratified by recruitment method. Independent samples *t* tests and chi-square tests of independence were used to investigate whether or not these measures differed significantly by recruitment method.

#### Costs

The direct research-related cost per completed survey was calculated for each recruitment method. For social media-based recruitment, cost per completed survey was calculated as follows: ([pilot ad cost + pilot-testing respondent incentives] + [recruitment ad cost + respondent incentives])/number of completed surveys as a result of Facebook approaches. For the clinical sample, cost per completed survey was calculated as follows: (respondent incentives + research assistants’ salaries)/number of completed surveys as a result of clinic approaches. Investigator costs were not included as the study team developed and deployed the campaigns as a group and focused on the Web-based and clinical efforts in a consistent fashion across both modes.

## Results

### Recruitment

**Figure 2:**
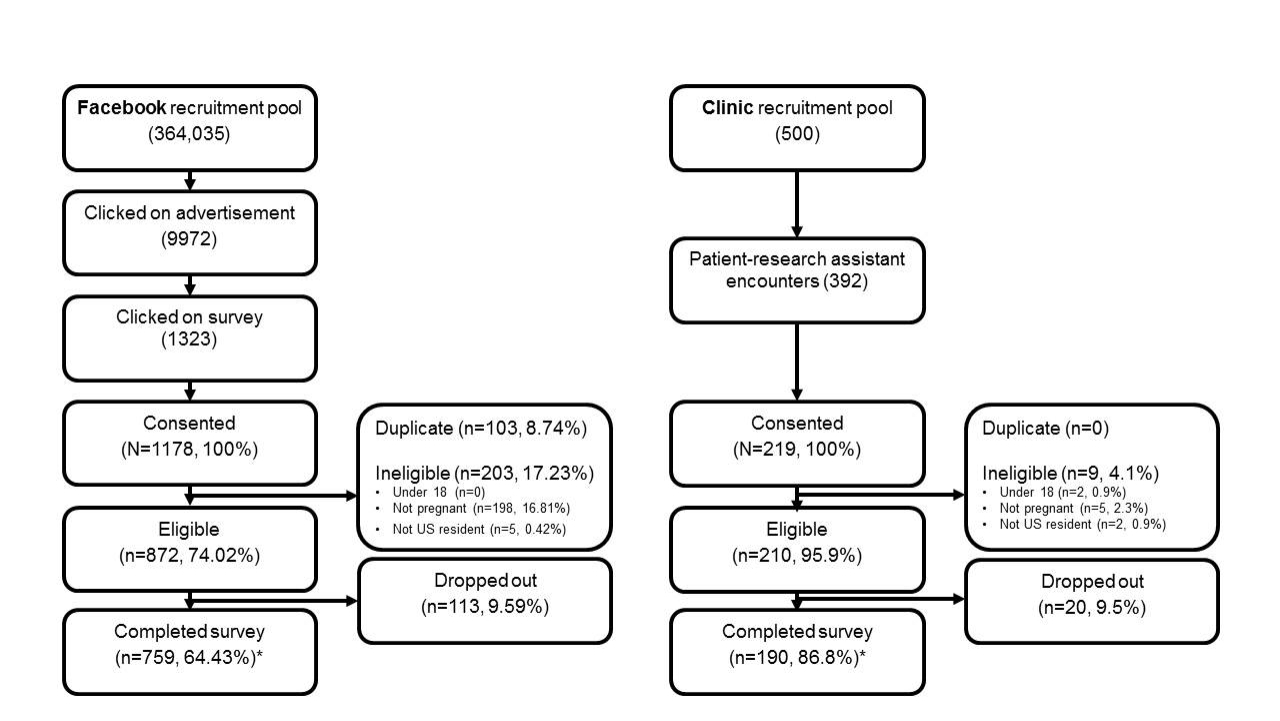
Flow diagram of inclusion, exclusion, and dropout of pregnant women recruited via social media–based compared to clinic-based approaches.

A flow diagram comparing social media-based with clinical recruitment is presented in Figure 2. Facebook ads were shown on 364,035 users’ newsfeeds over the 4-week campaign period. There were 9972 clicks on the ads, resulting in 1323 entries to the survey’s webpage and 1178 respondents who consented to participate. Among consenting respondents recruited via Facebook, 74.02% (872/1178) met eligibility criteria and 64.43% (759/1178) completed the survey.

During in-person clinical recruitment, approximately 500 unique pregnant patients were seen in clinic. Among the patients consenting to participate, 95.9% (210/219) met eligibility criteria and 190 86.8% (190/219) completed the survey.

### Demographics

A higher proportion of subjects recruited through social media self-identified as African American (29.4%, 207/705 vs 11.2%, 20/179; *P*<.001; Table 1) and reported annual household incomes <US $30,000 per year (56.8%, 409/720 vs 25.8%, 47/182; *P*<.001). A significantly lower proportion of those recruited through social media had earned a college degree (21.3%, 153/717 vs 62.3%, 114/183; *P*<.001) and were married or in a domestic partnership (45.7%, 330/722 vs 72.1%, 132/183; *P*<.001). With respect to health status, a higher proportion of those recruited through social media were in the first trimester of pregnancy (18.6%, 135/726 vs 10.4%, 19/183; *P*<.001) and rated their own health as fair or poor with greater frequency (22.2%, 160/722 vs 8.2%, 15/183; *P*<.001).

### Cost

**Figure 3:**
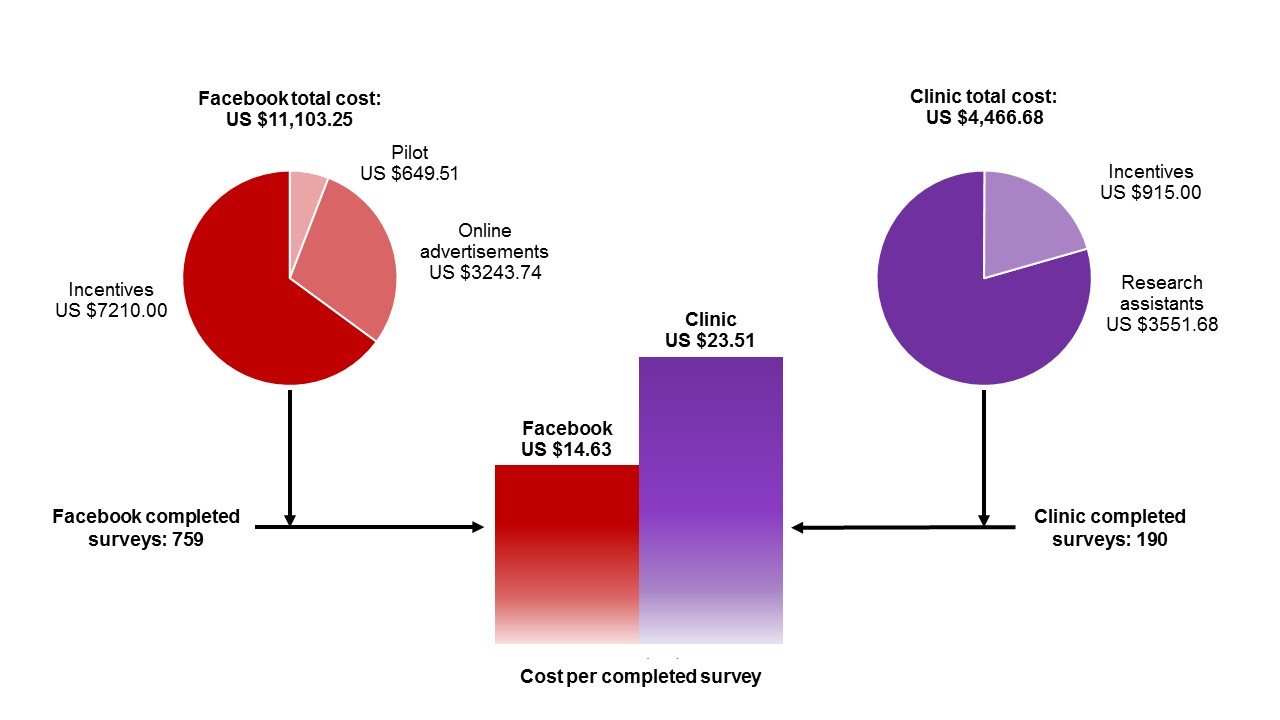
Cost per completed survey among pregnant women recruited via social media–based compared to clinic-based approaches.

The total cost of social media-based recruitment was calculated by adding the pilot-testing cost (pilot ads US $494.51 + pilot incentives US $155.00) and the final campaign cost (ads US $3243.74 + incentives US $7210.00), which totaled US $11,103.25 (Figure 3). Dividing the total cost by the total number of surveys completed as a result of Facebook approaches (n=759), the cost per completed survey was determined to be US $14.63.

The total cost of recruiting the clinical sample was calculated by adding expenditures on research assistants’ salaries (US $3551.68) and on participant incentives (US $915.00), which totaled US $4466.68. Dividing the total cost by the total number of surveys completed as a result of in-clinic approaches (n=190), the cost per completed survey was determined to be US $23.51.

**Table 1:** Characteristics of pregnant women recruited via social media-based versus clinic-based approaches.

Variable		Social media-based recruitment (n=759)	Clinic-based recruitment (n=190)	*P* value
Age in years, mean (SD)^a^		27.3 (5.2)	29.9 (4.7)	<.001
**Gestational age, n ( %)**		n=726	n=183	
	<14 weeks	135 (18.6)	19 (10.4)	
	14-27 weeks	276 (38.0)	54 (29.5)	
	>28 weeks	315 (43.4)	110 (60.1)	<.001
**Race, n (%)**		n=705	n=179	
	White only	283 (40.1)	130 (72.6)	
	African American only	207 (29.4)	20 (11.2)	
	Multiracial or other	215 (30.5)	29 (16.2)	<.001
**Education, n (%)**		n=717	n=183^b^	
	Did not complete high school	42 (5.9)	5 (2.7)	
	High school diploma	220 (30.7)	20 (10.9)	
	Associate’s degree or some college	302 (42.1)	44 (24.0)	
	College graduate	153 (21.3)	114 (62.3)	<.001
**Relationship status, n (%)**		n=722	n=183	
	Single	73 (10.1)	8 (4.4)	
	In a relationship	319 (44.2)	43 (23.5)	
	Married or domestic partnership	330 (45.7)	132 (72.1)	<.001
**Household income, US $, n (%)**		n=720	n=182	
	<30,000	409 (56.8)	47 (25.8)	
	30,000-60,000	231 (32.1)	37 (20.3)	
	>60,000	80 (11.1)	93 (53.9)	<.001
**Rating of own health, n (%)**		n=722	n=183	
	Excellent or very good	276 (38.2)	106 (57.9)	
	Good	286 (39.6)	62 (33.9)	
	Fair or poor	160 (22.2)	15 (8.2)	<.001
**Region^c^, n (%)**		n=563^b^		
	Northeast	32 (5.7)		
	Midwest	342 (60.8)		
	South	114 (20.3)		
	West	75 (13.3)		

^a^Social media-based recruitment n=750.

^c^Sum of percentages for subpopulation is within 0.1 of 100.0 due to rounding error.

^c^The region was not asked in clinic-based recruitment as the participants were recruited from a clinic in the Midwest.

## Discussion

### Principal Findings

Our findings indicate that Web-based recruitment may be a feasible approach to recruit a large, diverse sample of pregnant women at low cost relative to traditional, clinic-based recruitment. Over similar periods of time, the social media-based approach generated 5 times as many pregnant women for survey research compared to clinic recruitment. Women recruited through social media were also more demographically diverse than clinic recruits, and social media-based recruitment cost less per completed survey. Given that participants reported early gestational ages and were widely distributed geographically, it is unlikely that a similar cohort could have been recruited in 1 month using traditional in-clinic methods, even at a higher cost.

Recruitment of racially and ethnically diverse samples of pregnant women for research has been identified as a priority by the Division of Reproductive Health at the Centers for Disease Control and Prevention [16]. Adverse pregnancy outcomes, including severe maternal morbidity and mortality, are disproportionally higher among many minority populations [16,17]. At the same time, racially and ethnically diverse populations have been historically underrepresented in clinical research efforts for reasons including failure on behalf of researchers to reach minorities [18]. Furthermore, the association between socioeconomic disadvantage and poorer maternal and fetal pregnancy outcomes has long been established [19,20], as has the large and persistent association between lower educational attainment and worse health [21]. Our findings support the feasibility of social media to access vulnerable populations who may be harder to reach through traditional, clinic-based approaches.

Lower costs for social media-based recruitment (US $14.63 per completed survey) versus clinic-based recruitment (US $23.51 per completed survey), in combination with faster recruitment over time, suggest that social media-based recruitment offers a so-called dominant strategy—the process is more favorable and costs less. Importantly, it is possible that recruitment goals for either method could be met in shorter time with larger incentives or at cheaper cost over a longer period. Post hoc consideration of cost data for clinic recruitment revealed that the *rate* of accruing new subjects decreased over time, while the *cost* of accruing new subjects increased over time—indicating decreasing efficiency. In contrast, likely due to the exponentially larger pool of eligible participants on Facebook, the rate of subject accrual and cost were linear over time, which may make projections of recruitment through social media more predictable. While time-to-saturation of clinical recruitment pools may vary based on the characteristics of different patient populations, our findings may guide other researchers in estimating their own costs for reaching recruitment goals.

Despite these advantages, recruitment through social media has important disadvantages. This recruitment approach involves snowball sampling within established social networks; therefore, it may not be ideal for surveys trying to measure diversity of attitudes, perceptions, or experiences in care. For instance, snowball sampling likely explains the overrepresentation of those from the Midwest in the Facebook recruitment population. Fortunately, an inherent capability of the Facebook approach is that ad performance and cost can be monitored and adjusted in real time, which requires minimal time or technical expertise. Investigators can engage in purposive sampling by targeting ads to underrepresented users based on changing targets for demographic characteristics or interests as the study progresses.

### Limitations

Limitations of the study design should be considered when interpreting our findings. While clinic-based recruitment may have produced a more diverse sample in different or more numerous clinical settings, recruited participants will largely reflect the sample of patients served in a specific clinical setting. The Internet, in contrast, offers an inherently broader, more diverse recruitment pool and the potential advantages of targeting specific individuals or groups. Next, eligibility was assessed by self-report. It is unclear whether reliability of self-report differs between Web-based versus in-person recruitment settings, and comparative assessment of the quality of data obtained through social media is an important direction for future research [22]. Because of failure to meet eligibility criteria, there was a notably higher dropout rate in the Facebook sample. In future work, Facebook ads could be edited to more clearly reflect requirements for participation. Finally, there were also some indirect research costs, which were unclear in terms of their implications and are not included in reported estimates. These include 6 phone calls with Facebook and the labor required to train clinic clerks and research assistants, which required approximately 10 hours of the authors’ time in total and were split across the recruitment modalities.

### Conclusions

Successful survey research designed to improve the care of pregnant women requires the recruitment of diverse, adequately powered samples of participants. There are significant barriers to achieving this outcome with traditional, in-person recruitment, and the extant literature offers little comparative guidance regarding recruitment methods. Our head-to-head comparison of social media-based versus clinic-based recruitment of pregnant patients for survey research suggests that the use of a social networking platform is a feasible, inexpensive, and efficient approach to recruiting a large, diverse sample of pregnant women for survey research.
